# Anti-Müllerian hormone and progesterone levels in human follicular fluid are predictors of embryonic development

**DOI:** 10.1186/s12958-019-0492-9

**Published:** 2019-06-19

**Authors:** Yvonne O’Brien, Mary Wingfield, Lynne C. O’Shea

**Affiliations:** 1 Merrion Fertility Clinic, 60 Mount Street Lower, Dublin 2, Ireland; 2 National Maternity Hospital, Holles St, Grand Canal Dock, Dublin 2, Ireland; 3 UCD School of Medicine and Medical Science, Health Sciences Centre, University College Dublin, Belfield, Dublin 4, Ireland

**Keywords:** Anti-Mullerian hormone, Progesterone, Follicular fluid, Oocyte competence

## Abstract

**Background:**

Human follicular fluid is an intricate biological fluid contributing to the developing oocyte microenvironment. Accumulating evidence suggests that sex hormones present in follicular fluid (FF) may play an important role in regulating oocyte developmental potential. The aim of this study was to determine if anti-Müllerian hormone (AMH) and progesterone (P4) levels in FF are correlated with oocyte quality as defined by subsequent embryonic development.

**Methods:**

This was a prospective cohort study of 88 women undergoing IVF/ICSI at a university associated fertility clinic. Follicular fluid was collected from the first follicle aspirated at the time of oocyte retrieval. The corresponding oocyte was individually cultured in order to track its developmental outcome. FF-AMH and P4 concentrations from follicles where the oocyte fertilised normally and developed into a blastocyst on day 5 (Group 1: BLAST, *n* = 23) were compared with FF from follicles where the oocyte fertilised normally but failed to reach blastocyst stage by day 5 (Group 2: FERT, *n* = 19). No significant differences were observed between the two groups in terms of maternal age, body mass index, previous live births, previous pregnancy loss, number of antral follicles, number of oocytes recovered, IVF:ICSI ratio or percentage of recovered oocytes that fertilised.

**Results:**

FF-AMH and P4 levels were significantly increased in Group 1: BLAST compared to Group 2: FERT (*P* = 0.007 and *P* = 0.013 respectively). Twenty-one FF samples had an AMH level > 15 pmol/L, of which 17 related to oocytes that progressed to blastocyst stage, providing a positive prediction value (PPV) of 76.96%. Eleven FF samples had a P4 level > 60 mg/ml, of which 10 progressed to blastocyst stage, providing a PPV of 90.99%. Six samples had an AMH level > 15 pmol/L and a P4 level > 60 mg/ml, of which 100% progressed to blastocyst stage, providing a PPV of 96.83%.

**Conclusions:**

FF-AMH and P4 levels from individual follicles can accurately predetermine subsequent embryonic development. Combining follicular fluid analysis with routine morphological assessment, could allow for a more accurate and sensitive method of determining embryonic developmental competence.

## Background

We know that only a small number of the oocytes retrieved in an assisted reproduction technology (ART) cycle have the potential to develop into a viable embryo resulting in a live birth [1]. Despite current methods of embryo selection, including morphological assessment, time-lapse imaging and preimplantation genetic screening, the ability to predetermine an oocyte’s developmental potential remains a major obstacle to overcome [2]. As a result, all oocytes retrieved are inseminated, resulting in supernumerary embryos. In some cases, multiple embryos are transferred per treatment cycle with a view to increasing pregnancy rates. The subsequent increase in multiple pregnancy rates occurs at the expense of an increased risk of complications both for the mother and the offspring [3]. Furthermore, in oocyte vitrification cycles performed for the purposes of fertility preservation the optimal number of oocytes required for success has yet to be determined. As such, further understanding the follicular mechanisms regulating the acquisition of oocyte competence is an important goal. In addition, the discovery of an accurate, non-invasive and cost-effective predictive test of the development potential of an oocyte could have major impacts on the field of assisted reproduction.

The follicular fluid (FF) hormone microenvironment is known to regulate oocyte maturation, oocyte quality and subsequent embryonic development [4]. However, there is conflicting data in the literature concerning the relationship between the key FF hormones, progesterone (P4) and anti-Mullerian hormone (AMH), and reproductive outcome following ART. P4 is an intra-follicular steroid that plays critical roles in resumption of oocyte meiosis, fertilization, embryonic development, implantation and maintenance of pregnancy [5–7]. Several studies determined high FF P4 concentrations to be predictive of subsequent implantation [8–11]. However, additional studies have shown that oocytes from follicles with high FF P4 were associated with post-mature oocytes; displaying abnormal fertilisation with multi-pronucleation [12].

There is also conflicting evidence regarding the correlation of FF AMH concentrations with oocyte developmental competence. AMH is a glycoprotein dimer of the transforming growth factor-B super family and is an important regulator of follicle development [13]. It is produced independently of follicle-stimulating hormone (FSH) by the granulosa cells of the preantral and small antral follicles [14, 15]. AMH has been shown to be a very good marker of ovarian reserve, while several studies have demonstrated serum AMH to be an indicator of oocyte and embryo quality during COS [16]. However, the relationship between FF-AMH and oocyte developmental competence remains to be determined. Kede-Dickman et al. demonstrated that follicular fluid AMH concentrations were significantly higher in immature oocytes in comparison to MII oocytes [17]. Some investigators [18] could not find a correlation between FF AMH levels and fertilization rates or embryo morphology, whereas others [19, 20] observed a better prognostic value for fertilization outcome. In another study of pooled samples, the concentrations of AMH in FF were significantly higher in the group of women who became pregnant in the corresponding treatment cycle than in those who did not conceive [21].

The main objective of our study was to interrogate the utility of two well-known sex hormones, AMH and P4, in FF as potential biomarkers of successful development of a fertilised oocyte to a blastocyst stage embryo.

## Methods

### Study protocol

The sample size was determined by firstly estimating the minimum change needed to give a difference in P4/AMH follicular fluid concentration. Using results from a previously published paper [9, 18], it was determined that a 15% difference would be the desired minimum change, with a standard deviation of 20%. This gave a difference to be detected of 1.3 SD units (20/15). Using a confidence interval of 95%, 16 participants per group is necessary to provide a significant result. In order to account for data that is not normally distributed, we inflated the sample by 1.16 (Pitman). 16 × 1.16 = 18.56.

Patients undergoing IVF or ICSI cycles at Merrion Fertility Clinic (Dublin) were recruited and provided written informed consent. Patients suffering from PCOS were excluded from the study.

Patients either received a standard GnRH agonist (Buserelin/ Suprecur/ Deceapeptyl) regime or a standard antagonist regime (Orgalutran). Recombinant FSH (Gonal F/Puregon) or human menopausal gonadotrophin (Menopur) stimulation was initiated once down-regulation was confirmed via transvaginal ultrasound and serum oestradiol measurements or on day 2 or 3 of the cycle for antagonist cycles. Final oocyte maturation was achieved using 5000–10,000 iu human chorionic gonadotrophin or an agonist trigger (Buserlin) when at least three follicles had reached a diameter of ≥17 mm. This was administered 36 h before transvaginal oocyte retrieval.

Oocytes were collected by transvaginal ultrasound-guided needle aspiration of the follicles under conscious sedation. To avoid sample contamination, FF was collected from the first follicle aspirated per patient. To minimise the collection of blood or media-contaminated samples, a midstream aspirate was collected for each patient. The follicle was then flushed, using Origio flushing media. If the oocyte was not present in the first aspirate this continued until an oocyte was retrieved from that follicle, this occurred in only two patients.

FF samples were centrifuged at 2000 *g* for 5 min, the supernatant was collected and stored at − 80 degrees Celsius until assayed. After oocyte retrieval, all oocytes were washed with Quinn’s Advantage™ Medium with HEPES. For IVF, oocytes were placed in the insemination medium (Quinn’s Advantage Protein Plus Fertilisation Medium) 4–6 h before insemination with 100,000 motile spermatozoa per millilitre of medium. For ICSI, oocytes were initially placed into fertilisation medium (Quinn’s Advantage Protein Plus Fertilisation Medium). ICSI was performed 3 to 5 h after oocyte retrieval and the injected oocytes were then placed into cleavage medium (Quinn’s Advantage Cleavage Medium). Fertilisation was assessed 16 to 18 h after insemination or injection by the presence of 2 pronuclei and a second polar body. The fertilised oocytes were maintained in the culture medium (Quinn’s Advantage Cleavage Medium) until day three. If cultured until blastocyst stage, the embryos were transferred to blastocyst medium on day 3 (Quinn’s Advantage Blastocyst Medium).

**Fig. 1:**
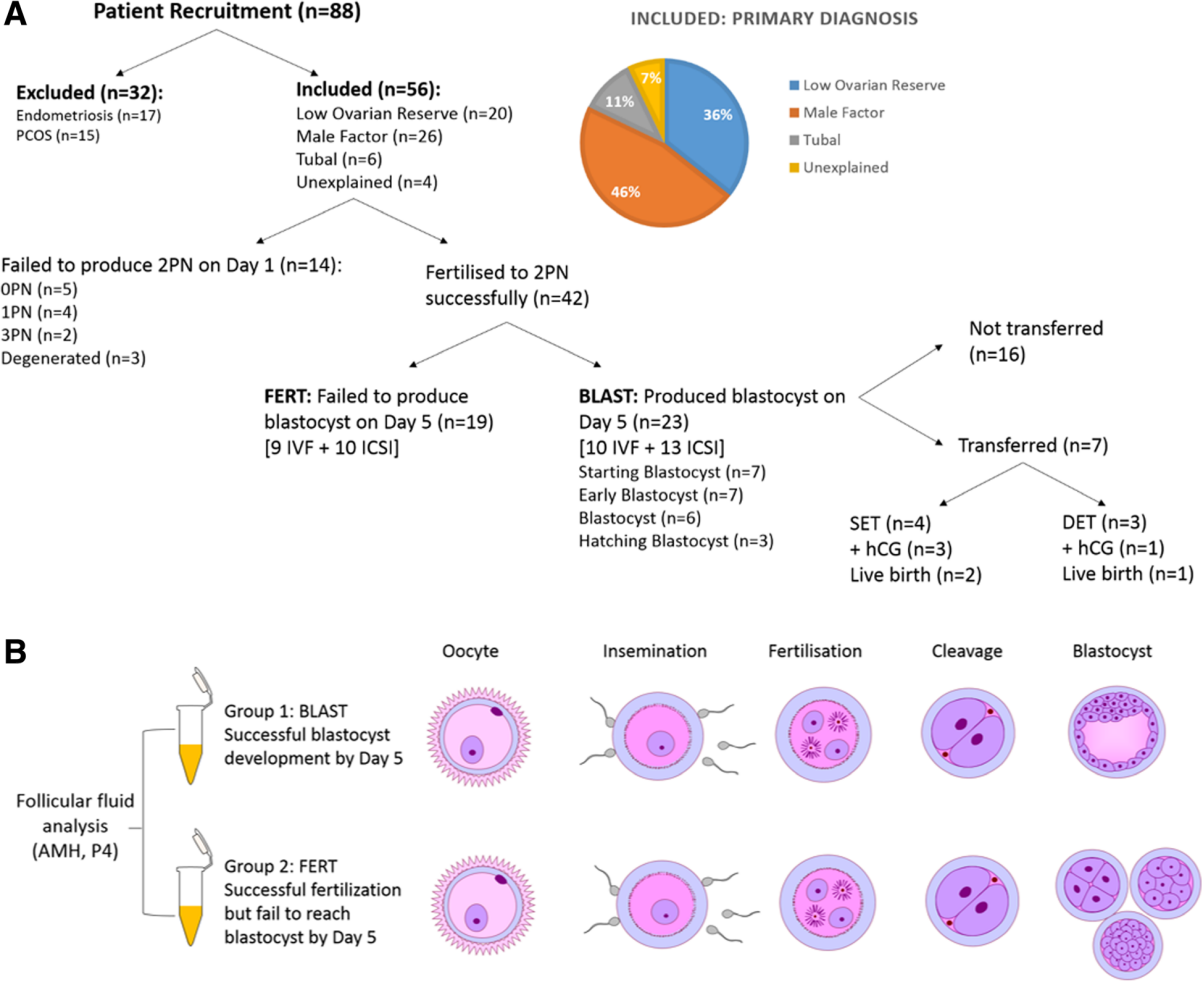
Correlation of follicular fluid hormones with embryonic development. **a** Schematic representation of patient selection, sample inclusion, fertilisation and embryo development. SET: Single Embryo Transfer; DET: Double Embryo Transfer; PN: Pronucleus; +hCG: positive human chorionic gonadotropin (pregnancy) test. **b** Diagrammatic representation of differential oocyte quality and subsequent embryonic developmental competence. Follicular fluid used in the current study was isolated from the first follicular aspiration, during oocyte recovery at a private fertility clinic. The developmental outcome of each oocyte was recorded based on whether they reached blastocyst on Day 5 of in vitro embryo culture. Samples were classified as either fertilising and developing to blastocyst (BLAST) or fertilising but failing to develop to blastocyst (FERT). Corresponding follicular fluid was evaluated for anti-Mullerian hormone (AMH) and progesterone (P4) protein levels. This figure was prepared using the Biomedical PPT toolkit suite (www.motifolio.com)

As part of a larger study, 88 FF samples were collected. None of the oocytes retrieved were immature – this may be due to the fact that the oocyte we collected was from the first follicle aspirated, and generally the largest follicle. None of the patient cycles selected resulted in total fertilisation failure. Samples were selected for analysis in this study based on the developmental competence of the oocyte i.e. follicular fluid from follicles where the oocyte fertilised and developed into a blastocyst as observed on day 5 (Group 1, BLAST; *n* = 23) and follicular fluid from follicles where the oocyte fertilised but failed to reach blastocyst stage by day 5 (Group 2, FERT; *n* = 19), as outlined in Fig. 1. In line with standard oocyte assessment in the ART laboratory, the two populations of oocytes were morphologically indistinguishable from each other prior to insemination and fertilisation.

### Follicular fluid analysis

FF AMH levels were measured using enzyme-linked immunosorbent assay (ELISA) (Immunotech Beckman Coulter, Marseille, France). The measurement range was 0.14–21 ng/ml. Intra- and inter-assay coefficients of variation were 12.3 and 14.2%, respectively, with a sensitivity of 0.14 ng/ml. FF progesterone levels were measured using ELISA (ADI-900-011; EnzoLifeSciences). Follicular fluid was diluted by a factor of 10 ^6^. The measurement range was 15.62–500 pg/ml. Intra- and inter-assay coefficients of variation were 7.6 and 6.8%, respectively, with a sensitivity of 8.57 pg/ml.

### Definition and predictive values of AMH and P4 groups

The cut off for defining AMH threshold concentration corresponded to the round value of the 50th percentile (> 15 pmol/L). The cut off for defining P4 threshold concentration corresponded to the round value of the 75th percentile (> 60 pmol/L). Sensitivity (if you test many blastocysts, the percentage that will have a positive test), specificity (if you test many embryos that fail to reach blastocyst, the percentage that will have a negative test) and prevalence (our test population contained 54% blastocyst samples) were used to calculate positive predictive values and negative predictive values. For AMH the following calculations were used: prior odds = prevalence/(100-Prevalence) = 1.174; likelihood ratio = sensitivity/(100-specificity) = 2.846; posterior odds = prior odds x likelihood ratio = 3.341; posterior probability = posterior odds/(1 + posterior odds) = 0.7696. For P4 the following calculations were used: prior odds = prevalence/(100-Prevalence) = 1.174; likelihood ratio = sensitivity/(100-specificity) = 6.8; posterior odds = prior odds x likelihood ratio = 7.983; posterior probability = posterior odds/(1 + posterior odds) = 0.8887. For AMH and P4 combined the following calculations were used: prior odds = prevalence/(100-Prevalence) = 1.174; likelihood ratio = sensitivity/(100-specificity) = 26; posterior odds = prior odds x likelihood ratio = 30.522; posterior probability = posterior odds/(1 + posterior odds) = 0.9683.

### Data management and statistical analysis

All data was entered prospectively on a customised excel database. Access to the database was password protected and the computer was also password protected. Each patient was assigned a study number. Shapiro-Wilk test was used to determine normal distribution. Data was analysed with SPSS software version 21.0 (SPSS, Chicago, IL, USA). Data were compared by independent-samples t-test, Welch’s t-test with Bonferroni correction or chi-square test, as appropriate. The result was considered as significant when the *P* value was < 0.05. Predictive values calculated from sensitivity, specificity and prevalence were calculated using GraphPad Prism.

## Results

### Clinical characteristics of study subjects and outcomes of controlled ovarian stimulation

To determine whether the clinical characteristics of the two groups were possible confounding factors, we compared these parameters, as presented in Table 1, between Group 1 (BLAST, *n* = 23) and Group 2 (FERT, *n* = 19). No significant differences were observed between the two groups in terms of maternal age, body mass index, previous live births, previous pregnancy loss, number of antral follicles, number of oocytes recovered, IVF:ICSI ratio or percentage of recovered oocytes that fertilised.

**Table 1:** Patient characteristics and follicular fluid profile corresponding to embryonic development

	Group 1: BLAST (n = 23)	Group 2: FERT (n = 19)	*P-value*
Maternal Age (years)	35.81 ± 3.76	36.05 ± 3.76	0.84
Body Mass Index	23.81 ± 3.06	22.85 ± 2.77	0.29
Oocytes collected	9.35 ± 4.18	9.25 ± 4.38	0.94
Normally fertilized (%)	67.83 ± 18.58	70.59 ± 19.73	0.63
Previous livebirths	0.31 ± 0.55	0.25 ± 0.55	0.73
Previous pregnancy losses	0.31 ± .47	0.20 ± 0.41	0.42
Antral Follicle Count	16.08 ± 9.20	12.0 ± 8.16	0.14
Serum AMH (pmol/L)	19.50 ± 18.02	18.10 ± 18.53	0.81

Values are given as mean ± standard deviations

Statistically significant, *P* < 0.05

### Correlation of follicular fluid AMH and P4 levels with embryo quality

**Fig. 2:**
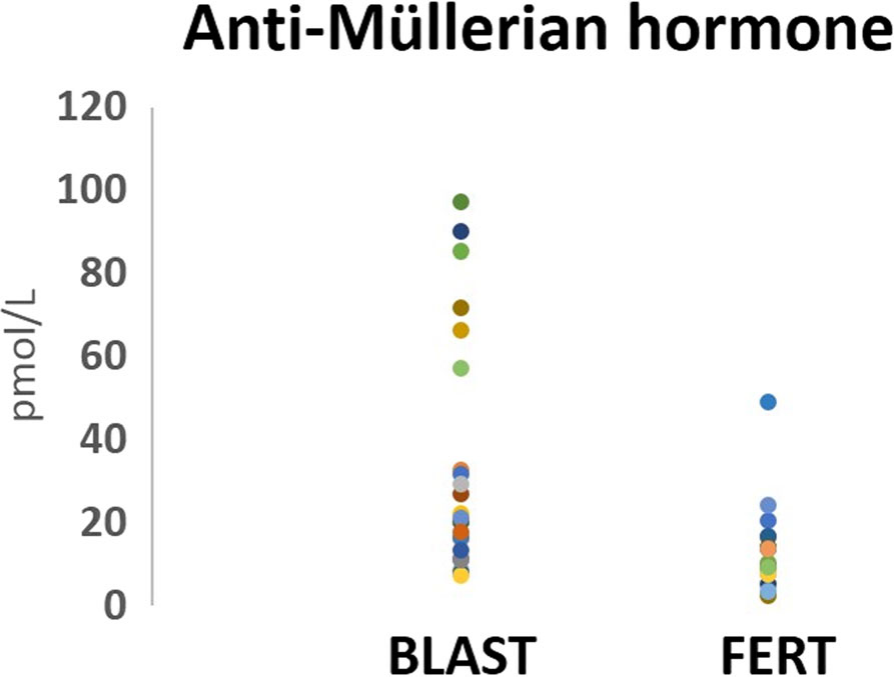
Levels of anti-Mullerian hormone in follicular fluid. The scatter plot represents the follicular fluid anti-Mullerian hormone (AMH) levels, in group 1 (BLAST, *n* = 23): follicles containing oocytes that develop to blastocyst stage by day 5 and group 2 (FERT, *n* = 19): follicles containing oocytes that fertilised but failed to reach blastocyst stage by day 5

**Fig. 3:**
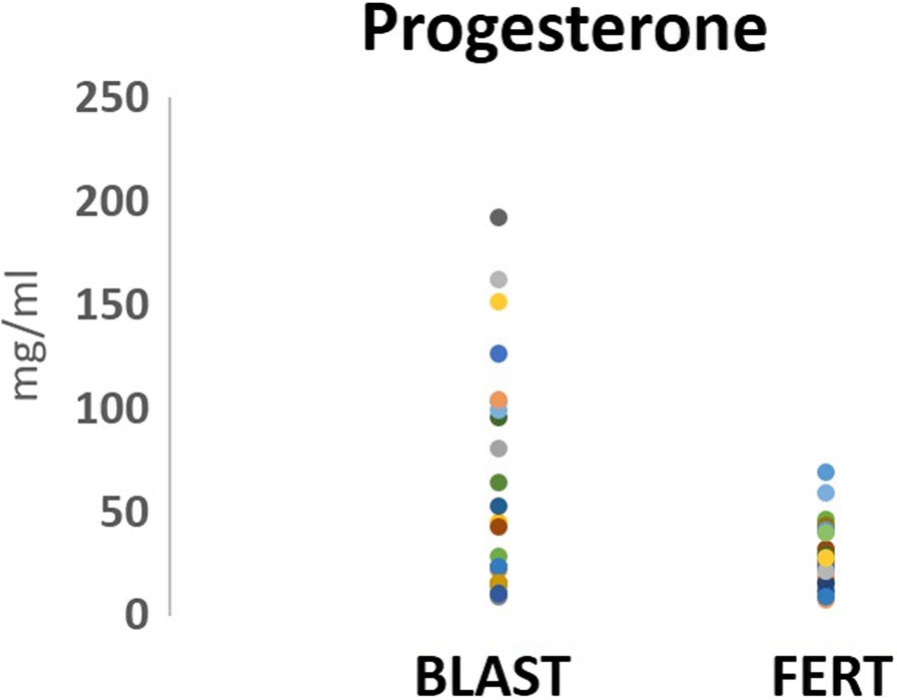
Levels of progesterone in follicular fluid. The scatter plot represents the follicular fluid progesterone levels, in group 1 (BLAST, *n* = 23): follicles containing oocytes that develop to blastocyst stage by day 5 and group 2 (FERT, *n* = 19): follicles containing oocytes that fertilised but failed to reach blastocyst stage by day 5

To evaluate the possibility of using key reproductive hormones as potential indicators of an oocytes developmental potential, FF levels of AMH and P4 were quantified and compared between the ‘BLAST’ (*n* = 23) and ‘FERT’ (*n* = 19) groups. FF AMH levels were significantly increased (*P =* 0.007) in the ‘BLAST’ group (33.13 ± 28.83 pmol/L) compared to the ‘FERT’ group (13.6 ± 12.3 pmol/L) (Table 2, Fig. 2). Similarly, FF P4 levels were significantly higher (*P* = 0.013) in the ‘BLAST’ group (59.76 ± 54.38 mg/ml) compared to the ‘FERT’ group (29.25 ± 22.4 mg/ml) (Table 2, Fig. 3).

**Table 2:** Follicular fluid levels of anti-Mullerian hormone and progesterone

	Group 1: BLAST (n = 23)	Group 2: FERT (n = 19)	*P-value*
Follicular Fluid AMH (pmol/L)	33.07 ± 29.20	13.6 ± 12.3	0.007
Follicular Fluid Progesterone (mg/ml)	62.5 ± 55.3	29.25 ± 22.4	0.013

Values are given as mean ± standard deviations. Statistically significant, *P* < 0.05

*AMH* anti-Mullerian hormone, *P4* progesterone

Twenty-one FF samples had an AMH level > 15 pmol/L, of which 17 related to oocytes that progressed to blastocyst stage; thus providing a sensitivity rate of 74%, a specificity rate of 74% and a prevalence rate of 54% (Table 3). This provides a positive prediction value (PPV) of 76.96%, leaving a 23.04% chance of a false positive. A negative prediction value (NPV) of 70.8% was obtained (i.e. if the sample fertilised but does not reach blastocyst there is a 70.8% chance of a negative test result), leaving a 29.2% chance of a false negative.

**Table 3:** Correlation between follicular fluid hormone concentration and embryonic development

Classification	BLAST (n)	FERT (n)	Positive Prediction Value	Negative Predication Value
Anti-Mullerian hormone level > 15 pmol/L Sensitivity: 43%; Specificity: 95%	17	4	76.96%	70.80%
Progesterone level > 60 mg/ml Sensitivity: 74%; Specificity: 74%	10	1	90.99%	58.67%
AMH level > 15 pmol/L + P4 level > 60 mg/ml Sensitivity: 26%; Specificity: 99.9%	6	0	96.83%	53.26%

*AMH* anti-Mullerian hormone, *P4* progesterone

Eleven FF samples had a P4 level > 60 mg/ml, of which 10 related to oocytes that progressed to blastocyst stage; thus providing a sensitivity rate of 43%, a specificity rate of 95% and a prevalence rate of 54% (Table 3). This provides a PPV of 90.99%, leaving a 9.01% chance of a false positive. A NPV of 58.67% was obtained, leaving a 41.33% chance of a false negative.

Six samples had an AMH level > 15 pmol/L and a P4 level > 60 mg/ml, of which 100% progressed to blastocyst stage; thus providing a sensitivity rate of 26%, a specificity rate of 99.99% and a prevalence rate of 54% (Table 3). This provides a PPV of 96.83%, leaving a 3.17% chance of a false positive. A NPV of 53.26% was obtained, leaving a 46.74% chance of a false negative.

## Discussion

The quality of the embryo is an important predictor of ART treatment success, as high quality embryos lead to a high pregnancy ratio [22]. The quality of oocytes obtained during IVF procedures varies considerably. Whilst most mature oocytes are amenable to fertilisation, only half of those fertilised complete embryonic development and even fewer implant [23]. As a result, many ART cycles fail to produce developmentally competent embryos which are suitable for transfer and which are capable of resulting in successful pregnancies.

In the present study we show, for the first time, that both FF-AMH and P4 levels from individual follicles can accurately predetermine subsequent embryonic developmental competence. These results suggest that the regulation of these hormones, within the follicular microenvironment, play an important role in determining oocyte developmental competence. Of significance, we identified definitive AMH (> 15 pmol/L) and P4 (> 60 mg/ml) follicular fluid levels that could be used as an accurate, non-invasive and cost-effective predictive test of the developmental potential of an oocyte in a clinical setting.

To determine the significance of these observations it is necessary to place them in context of the procedures currently employed in clinical practice. At present, it is not possible to accurately predetermine an oocyte’s developmental potential prior to in vitro embryo production. Such a test would enable a reduction in supernumerary embryo production, thereby overcoming the ethical, legal, and storage implications of current human assisted reproduction practices. Furthermore, from the perspective of oocyte vitrification, a test to predict the developmental potential of an oocyte could have major consequences for clinical practice and counselling of patients. Rather than the current recommendations of proposing a minimum number of 8–10 metaphase II oocytes to vitrify as necessary to have a reasonable chance at achieving a pregnancy [24], it would be possible to provide an individualised treatment protocol to retrieve the optimal number of high quality oocytes per patient.

In females, AMH is exclusively produced by granulosa cells of healthy ovarian follicles [14, 25–27]. Across mammalian species, AMH expression starts at the onset of follicle recruitment [28], reaching its highest level in preantral and small antral follicles. This is followed by a decrease in AMH levels as the selected, FSH-dependent follicle progresses toward the preovulatory stage, with AMH absent in atretic follicles [29, 30]. As such, AMH is dynamically regulated in response to both folliculogenesis and follicle quality, which may account for previous conflicting studies correlating both high [18, 21] and low [31] AMH FF levels with oocyte developmental competence. Unlike previous studies, here we have circumvented the fertilisation step of the ART process, limiting our analysis to post-fertilisation developmental competence. This has enabled us to specifically correlate increased FF AMH levels to blastocyst development, independent of fertilisation success and methodology. This approach provides greater insight into the regulation of this important developmental window.

We also show that high levels of FF-P4 correlate with blastocyst development. This is concurrent with previous studies reporting high FF P4 concentrations to be predictive of subsequent implantation [8–11]. During oocyte maturation in vivo, P4 synthesis occurs in the granulosa cells due to stimulation by FSH; with progesterone production further increasing in preovulatory follicles due to differentiation of LH receptors, corresponding to the LH surge [32]. This means that during maturation the oocyte is subjected to increasing concentrations of P4. A previous study in bovine demonstrated that in vitro maturation of oocytes in the absence of P4, resulted in decreased embryonic developmental competence [33]. Of significance, in this study there was no effect on fertilization and cleavage stages of development – with decreased developmental competence only observed at blastocyst stage. This is in line with the results observed in our present study, suggesting that decreased FF-P4 secretion during oocyte maturation can have detrimental effects on oocyte quality as defined by subsequent embryonic development.

Previous studies focused predominantly on day 2 and day 3 cleavage stage embryos [20]. Our protocol involving blastocyst stage embryos is more in keeping with those studies reporting high FF-P4 concentrations to be predictive of subsequent implantation [8–10]. In addition, we studied FF from a single follicle and followed the development of its particular oocyte whereas others have used pooled FF [31]. It is postulated that assays from pooled follicular fluid may not reflect the microenvironment surrounding a specific oocyte [20].

## Conclusions

We have demonstrated that elevated AMH and P4 in FF is correlated to the ability of a fertilised oocyte to reach the blastocyst stage. We have identified, for the first time, definitive AMH and P4 FF protein levels that could be used as a biomarker array, to determine the developmental potential of an oocyte. Our significant findings prompt additional, larger-scale studies to evaluate further the definitive parameters and predictive potential of FF-AMH and P4 as non-invasive markers of oocyte competence in a clinical setting, including ART protocol. Combining follicular fluid analysis with routine morphological assessment, could allow for a more accurate and sensitive method of determining embryonic developmental competence.

## Data Availability

The datasets used and/or analysed during the current study are available from the corresponding author on reasonable request.

## Abbreviations

AMH: Anti-Müllerian hormone
ART: Assisted reproduction technology
BMI: Body mass index
ELISA: Enzyme-linked immunosorbent assay
FF: Follicular Fluid
FSH: Follicle-stimulating hormone
P4: Progesterone
